# Supplementary material for: Lessons from a systematic tracing process aimed to reduce initial loss to follow-up (ILTFU) among people diagnosed with tuberculosis (TB) in Cape Town, South Africa
Source: PLoS One. 2025 May 30;20(5):e0323943. doi: 10.1371/journal.pone.0323943 (PMC12124562; doi:10.1371/journal.pone.0323943)
Supplement: Attachment — Submitted filename: Response to Reviewers.docx [file pone.0323943.s002.docx]

Dear Editor

Thank you for the opportunity to revise our paper. We attended to the recommendations and provided detailed feedback to each comment on the table below.

| **Recommendations** | **Response** |
| --- | --- |
| 1) Please ensure that your manuscript meets PLOS ONE's style requirements, including those for file naming. | Noted, thank you. |
| 2) One of the noted authors is a group or consortium [LINKEDin study team]. In addition to naming the author group, please list the individual authors and affiliations within this group in the acknowledgments section of your manuscript. Please also indicate clearly a lead author for this group along with a contact email address. | We have listed all the authors who are part of the manuscript and removed the statement that related to a group. The authors of this manuscript are limited to the people listed. |
| 3) Please note that your Data Availability Statement is currently missing [the repository name and/or the DOI/accession number of each dataset OR a direct link to access each database]. If your manuscript is accepted for publication, you will be asked to provide these details on a very short timeline. We therefore suggest that you provide this information now, though we will not hold up the peer review process if you are unable. | We have amended the data availability statement to read: “The data set file is available from the Figshare database (accession number(s) CC BY 4.0. “<https://doi.org/10.6084/m9.figshare.27179805>” |
| 4) Please review your reference list to ensure that it is complete and correct. If you have cited papers that have been retracted, please include the rationale for doing so in the manuscript text, or remove these references and replace them with relevant current references. Any changes to the reference list should be mentioned in the rebuttal letter that accompanies your revised manuscript. If you need to cite a retracted article, indicate the article’s retracted status in the References list and also include a citation and full reference for the retraction notice. | We have reviewed and updated the references list. |
| Thank you to both reviewers for raising these important points, we appreciate the time taken to review and give advice on how to improve our manuscript**.** | |
| **Reviewer 1: Comments** | **Response** |
| 1)Abstract: • The last sentence of results isn’t clear standalone – see comment on related results section.  • The whole abstract is 340 words and was truncated in the review system. Instructions are that abstracts should not exceed 300 words. | Noted, we have revised the sentence.  We cut accordingly. The word count is now 300 words. |
| 2) Methods:  Line 79/80 with reference 6 – it is unclear what this refers to as the reference gives only a year and volume which is 12 editions of the journal with multiple articles. | Thank you, we have updated this reference to: <https://doi.org/10.1093/ofid/ofad648> |
| 3) Line 159 refers to a final extract ‘4 months after the end of the systematic tracing process’ but the abstract refers to ‘linkage outcomes five months after the end of the process.’ | Yes, the final data extract from the PHDC was done 4 months later. Thereafter, we verified and cleaned the data set, with the final outcomes concluded on the 5^th^ month. We have revised this for clarity. |
| 4) Results: While not a key result, for context it would be helpful to know what proportion the 427 identified constitute of all people in the PHDC records for this period. | The PHDC is organized in disease cascades e.g. TB (which we utilized), HIV, pregnancy etc. Researchers obtain approval to access the relevant cascade. We cannot provide the total number of people recorded in the PHDC during this period. There were 831 persons recorded in the TB cascade (had a TB diagnosis) during this period. |
| 5) Line 217-218 ‘Of the 107 people that required home-based tracing but could not be found, 77/107 (72%), linked to care (see Figure 3).’ This sentence perplexed me, as did the related sentence in the abstract (line 49).  Figure 3 finally clarified that the way you knew their status was through verification in PHDC post-intervention. Please make this clear in the text. | Thank you for the comment, we have clarified this in line 217-218 to ‘Of the 107 people that required home-based tracing but could not be found, 77/107 (72%), were later confirmed to be linked to care through the PHDC post-intervention (see Figure 3). |
| 6) The section and table (1) on Characteristics of people with TB who were traced and found vs not found could be strengthened by some simple statistical comparisons of the proportions reported. | Thank you for this suggestion. We have added a column in the table to show the p-value difference between groups tested for significance at 0.05. |
| 7) Discussion  Line 289 spelling error – patient-centred. | The error is corrected, thank you. |
| 8) line 291 spelling error – supported. | We corrected the error, thank you. |
| 9) line 208 spelling error – lost to follow-up. | We corrected the error, thank you. |
| 10) References: Reference 6 and 12 are incomplete/insufficient information. | We have updated reference 6 and checked reference 12, which appears complete. . |
| **Reviewer 2: Main Comments** | **Response** |
| 1) A line or two under “Setting” to explaining the current system (if any) to link individual records from diagnosis to treatment and follow up, would be helpful eg is there a unique identifier system in use and how is that working or not working? Given that one of the key findings and lessons learnt from this study is the need to improve the capturing of individual records accurately especially the individual contact details, it is worth understanding the existing system that could be strengthened and how. | We have added more information from line 108-116. |
| 2) Similarly Line 318-321 – I would suggest to consider adding improved linkage of individual person records eg through a unique identifier or similar, to the proposed efforts to strengthen linkage to TB care. | We have revised this sentence line337-342 “this study highlights key aspects within the TB program that can be targeted to improve linkage to TB, by improving the data capturing quality for accurate personal information which will be linked with their unique identifiers to strengthen linkage to care, real-time data availability; improved communication between healthcare workers and persons with TB and updating contact details at every healthcare visit. |
| 3) I suggest highlighting what the “true” ILTFU was after all the efforts done to contact PHC facilities and persons with TB (telephone and home visits) | Thank you, we have added this on line 229-231. |
| 4) Line 236 (Table 1) – comparison between 2 groups ie “ILTFU found” and “ILTFU not found” I am not a biostatistician but I think some analytical stats would be helpful to understand if there are differences between these two samples. | We compared the two groups with categorical variables using a chi square test and added a column in the table to show the p-value difference between groups tested for significance at 0.05. |
| **Other Comments** | **Response** |
| 1)Line 59: “The Tuberculosis (TB) epidemic is a leading health crisis [1] with 10.6 million people diagnosed with TB in 2022; 23% of whom were in Africa” This should read “…an estimated 10.6 million…” | We corrected this, thank you. |
| 2) Line 72: “aimed to evaluate the yield, effort required and lessons learned from a systematic follow up process to find people who were ILTFU “ I suggest adding ‘document’ to read “… and document lessons learned…” | Thank you for the suggestion which we have used. |
| 3) Line 181-183 “Of these, 21/427 (5%) were excluded from the analysis because they were subsequently found not to have TB (based on subsequent TB test results showing a negative TB culture result).” 5% is a lot to have been excluded on the basis of a subsequent culture negative test. Is there data from other settings in South Africa and how does that compare with your study?  Are these persons initially diagnosed with TB and referred to PHC for treatment?  How are these persons excluded from the final TB notification data under the routine TB program? | Concern on Xpert false positivity in SA has been related to previous TB treatment history.  In a study in the same city (Cape Town), as high as 1 in 7 Xpert-positive retreatment patients were culture negative and potentially false positive (https://academic.oup.com/cid/article/62/8/995/2462404).  While this is an interesting track it is beyond the scope of this project. False positives need to be explored in terms of previous TB, timing since treatment, exposure and other factors but this is not the focus of this submissions  Yes, they initially were recorded as having a positive TB test. A referral to a PHC facility would happen if they were tested at a hospital and discharged, but would have a return date to collect their results if tested at a PHC facility.  Thank you, this is an important point. There are a number of reasons for their removal which can include an error in the diagnosis or a clinical decision. Our study was not designed to further investigate the changes in the TB cascade but limited improving linkage to TB care. |
| 4) Line 193-194 Only 11 (out of 219) eligible for tracing has a contactable phone number. That is another major finding given a mobile phone is more accessible now and individuals from informal settlements are likely to have a phone (number) rather than a physical address. Is this due to inaccurate recording of the phone numbers or persons with TB deliberately gave wrong numbers? If the latter, then there is need to understand the reasons why and address them. | Poor data capturing is another contributing factor to this problem as described in our findings. Our study is not able to comment beyond this since we did not collect data on why there are so few patients with contactable phone numbers.  We have highlighted this point in the discussion line 285-289: “The relatively low number of eligible people with contactable phone numbers even in a setting with relatively broad access to mobile phones may represent an ongoing challenge to high coverage of ILTFU interventions especially to the most socio-economically marginalised.” |
| 5) Line 221-222: Figure 3: Linkage to care outcomes for persons traced who were found and needed referred vs not found. Probably a typo in the title. Did you mean “needed referral”? | Yes, and thank you we have revised as suggested. |
| 6) Line 241-244 – sentence too long. Consider revising it. | Revised, thank you. |
| 7) Line 264-265: “In our study, we were only able to successfully contact 11/209 (5%) persons we needed to trace”. I suggest adding that you were able to successfully contact by telephone otherwise can be misconstrued as the only ones you managed to reach through tracing. | This is correct, we have revised the sentence. |
| 8) Line 265-267 “A previous study in the same setting found 64% of TB patients in hospitals had an updated contact number from what was captured in their patient records [5].”  Any reasons for this big difference between your study ie 5% vs 64% the other study in the same setting? I think this is worth discussing. | We have revised the sentence in line 281-285 as “A previous study in the same setting, only 36% of TB patients in hospitals had the same contact number from what was captured in their patient records [5], with people lost to follow-up perhaps representing an especially socio-economically marginal group with greater life instability.” |
| 9) Line 289 – typo, should read “patient-centered…” | We corrected the error, thank you. |
| 10) The manuscript uses “patients” and “people with TB”. I suggest using the latter and for consistency. | We have corrected this and mainly used “people or persons” for consistency. |

We look forward to receiving your response.

Yours sincerely

Nosivuyile Vanqa

Email: nvanqa@sun.ac.za

Tel: +2721 938 9686
