# Supplementary material for: Lessons from a systematic tracing process aimed to reduce initial loss to follow-up (ILTFU) among people diagnosed with tuberculosis (TB) in Cape Town, South Africa
Source: PLoS One. 2025 May 30;20(5):e0323943. doi: 10.1371/journal.pone.0323943 (PMC12124562; doi:10.1371/journal.pone.0323943)
Supplement: Attachment — Submitted filename: Response_to_Reviewers_auresp_2.docx [file pone.0323943.s003.docx]

Dear Editor

Thank you for the opportunity to revise our paper. We attended to the recommendations and provided detailed feedback to each comment on the table below.

| **Recommendations** | **Response** |
| --- | --- |
| **Editor comments** | |
| 1) The manuscript deserves a significant revision of the 'Background." The two-paragraph Background does not rationalize the study. The background is not sufficiently rooted in the existing literature on contact tracing and pre-treatment lost-to-follow-up persons with tuberculosis. A more meaningful introduction must answer these basic questions: What did we study? Why was it important to study the phenomenon? What do we know from the existing scholarship? How has the country of study addressed the concerns through its policies and programs? What research gaps is the study contributing to fill? The authors would realize that the Discussion section of the manuscript would benefit from further revision when they revise the Background along the suggested lines. It would be best for the authors to address these deficiencies to get the paper to a publishable level. | The background has been completely revised.  The discussion has been revised as required and in relation to the revisions in the background.  The abstract has been revised to align with the revisions in the body of the manuscript. |
| 2) Furthermore, while the paper's title focuses on lessons from a systematic tracing process, the Results section of the abstract does not indicate any lessons. This is a fundamental omission! The authors need to revise the Results section of the abstract to include essential statistics and lessons learned. | We have substantially revised the abstract and added lessons learned into the results section of the abstract. |
|  | |
| **Reviewer 1: Comments** |  |
| 1) In response to item 3. The changes in abstract methods about timing (4 or 5 months) still lack clarity. If diagnoses were included up until 31/12/2020, but tracing started only 3 weeks after a person was not found to be linked in PHDC, then 31/05/2021 isn’t really 5 months after the ‘process’ concluded as tracing for the people last diagnosed would have started only in January. The sentence in methods (line 165) is improved. Consider changing ‘process concluded’ in the abstract – e.g. five months after the inclusion period? | The abstract has been substantively revised and this sentence has been deleted to prevent unnecessary confusion. |
| 2) Linked to the issue of period of intervention/study, duration of attempt to trace people was not clear. Figure 1 shows 2 attempts 7 days apart, and tracing started only 3 weeks after diagnosis, but was there a limit such that tracing attempts would only occur within 1-2 months after the lists were generated? A sentence at the end of the paragraph ending line 152 would be helpful, if word-count allows. | We have included a sentence to specify the time of the tracing activities to “All tracing attempts occurred between 3 and 6 weeks after the lists were generated” See lines 174 - 175. |
| 3) In response to item 6. Statistics have been added – the test name is ‘Pearson’s’ and it should be listed in the analysis section of the methods. The final p-value in Table 1 looked very surprising for these proportions and I think you will find it is 0.52. | We have added the Pearson’s test to the data analysis paragraph. It reads, “We used Pearson’s chi square test to determine if there were any statistically significant differences in demographic and clinical characteristics and linkage outcomes between those who linked and had not linked to care.” See lines 197 – 199. |
| 4) Minor errors or word changes to consider: a. Line 49-51: Accurate data captured in ‘real time’ at PHC facilities will improve records and the resources to can be focused on truly lost to follow-up people. b. Line 131 remove unnecessary comma and I suggest change ‘ago’ to ‘earlier’: generated a list of persons who had been diagnosed with TB three weeks earlier c. Line 224 add ‘were’: The majority (37/49;76%) of people with TB that we traced, found, and referred to care were linked to a TB treatment facility d. Line 227 add missing closing bracket: True loss to follow up was 30/107 (28%) among those not found and 12/49 (24%) among those found and referred to care (see Figure 3). e. Line 241-242 add a space between semi-colon and % figures in brackets. f. Line 245: I suggest you reference Figure 3 at end of the sentence. g. Reference 12 is not incomplete but it has ‘The’ immediately after the doi, which seems to be an error and should be deleted. | a) The entire abstract has been revised. The conclusion now contains more substantive recommendations, which has resulted in this sentence being removed. (See Lines 44-47).  b) Changed as per your suggestion (now line 153).  c) Changed as per your suggestion (now line 249).  d) Changed as per your suggestion (now line 252).  e) Changed as per your suggestion (now line 255 - 256).  f) Changed as per your suggestion and revised the sentence for clarity (now line 269).  g) Deleted as per your suggestion. |
| **Reviewer 2: Comments** | **Response** |
| 1) Lines 49-51: it appears there could be a missing word before “…the resources…” Accurate data captured in ‘real time’ at 50 PHC facilities will improve records and the resources to be focused on truly lost to 51 follow-up people. | The entire abstract has been revised. The conclusion now contains more substantive recommendations, which has resulted in this sentence being removed. See Lines 48- 52). |
| 2) Lines 56-62 Suggest using the latest (2024) report | The Global TB Report (2024) has been used throughout, and all statistics quoted have been updated as per this latest report. |
| 3) Lines 110-113 – suggest rewording People are typically first registered in a paper-based register and thereafter into the electronic TB treatment register at the facility, using the unique identification number “personal number/folder number”. | Revised as suggested. Now lines 132-135. |
| 4) Lines 164 correct typo, ‘c’ missing in care. | Changed, now line 173. |
| 5) Lines 242-245: check calculation or denominator. 1/30 is 0.3%. Review last part of the sentence ie “…those found and referred to care with (17%) of death rate.” | Changed, now line 268. |

We look forward to receiving your response.

Yours sincerely

Nosivuyile Vanqa

Email: nvanqa@sun.ac.za

Tel: +2721 938 9686
