# Supplementary material for: Lessons from a systematic tracing process aimed to reduce initial loss to follow-up (ILTFU) among people diagnosed with tuberculosis (TB) in Cape Town, South Africa
Source: PLoS One. 2025 May 30;20(5):e0323943. doi: 10.1371/journal.pone.0323943 (PMC12124562; doi:10.1371/journal.pone.0323943)
Supplement: Attachment — Submitted filename: Response_to_Reviewers_auresp_3.docx [file pone.0323943.s004.docx]

Dear Editor

Thank you for the opportunity to revise our paper. We attended to the comments and have provided feedback to each comment on the table below.

Please note that we have reviewed every reference in our reference list to ensure that it is complete and correct. We have not cited any papers that have been retracted.

| **Editor Comments** | **Response** |
| --- | --- |
| I must commend the authors for a significant improvement in the manuscript. We are almost there! Nonetheless, I have few vital points they need to address.  1. Introduction: The last paragraph of the introduction can improve if they author leverage some of the studies cited in the discussion and 3 other South African papers (see below after my comments) to rationalize the study and establish the knowledge gap on systematic tracing of ILTFU - yield, efforts required, and lessons learned.  2. Data analysis: Lines 200-202 indicates the authors "reflected on the activities and lessons learned from the systematic tracing." How was this reflection done? Who was involved, and for how long? Did you keep journals/ notes during the systematic tracing? It might be helpful to clarify the reflection process in the data collection and data analysis sub-sections of the methods.  3. Discussion: The authors should take a second look at the sentence in lines 295-296 and revise it to convey the intended meaning. | Thank You.   1. We have revised the last paragraph of the introduction and cited additional papers, including the 3 named by the editor. 2. We have added the detail details on our reflection process. See lines 194-200. 3. We have revised this sentence to clarify our intended meaning. It now reads, ‘Of those we traced and found at home, almost half had linked to care in the time between us checking the records at the health facility and us visiting them at home.’ Lines 303-304 |
|  | |

We look forward to receiving your response.

Yours sincerely,

Nosivuyile Vanqa

Email: nvanqa@sun.ac.za

Tel: +2721 938 9686
